# Can Essential Oils/Botanical Agents Smart-Nanoformulations Be the Winning Cards against Psoriasis?

**DOI:** 10.3390/pharmaceutics15030750

**Published:** 2023-02-23

**Authors:** Mohamed Ashraf, Hossam S. El-Sawy, Ghada M. El Zaafarany, Mona M. A. Abdel-Mottaleb

**Affiliations:** 1 Department of Pharmaceutics and Pharmaceutical Technology, Faculty of Pharmacy, Egyptian Russian University, Cairo 11829, Egypt; 2 Department of Pharmaceutics and Industrial Pharmacy, Faculty of Pharmacy, Ain Shams University, Cairo 11566, Egypt

**Keywords:** psoriasis, essential natural oils, botanical remedies, nano-delivery systems, novel pharmaceutical formulations

## Abstract

Although psoriasis remains one of the most devastating inflammatory disorders due to its huge negative impact on patients’ quality of life, new “green” treatment approaches still need to be fully explored. The purpose of this review article is to focus on the utilization of different essential oils and active constituents of herbal botanical origin for the treatment of psoriasis that proved efficacious via both in vitro and in vivo models. The applications of nanotechnology-based formulations which displayed great potential in augmenting the permeation and delivery of these agents is also addressed. Numerous studies have been found which assessed the potential activity of natural botanical agents to overcome psoriasis. Nano-architecture delivery is applied in order to maximize the benefits of their activity, improve properties, and increase patient compliance. This field of natural innovative formulations can be a promising tool to optimize remediation of psoriasis while minimizing adverse effects.

## 1. Introduction

Psoriasis is a common, yet debilitating, immune-mediated inflammatory condition. It usually appears in the form of reddened, raised lesions or “plaques” on the skin, which may be covered with silver or white-colored scales. Individuals at risk of developing psoriasis often have genetic polymorphisms affecting genes that are involved in the adaptive and innate immune systems and/or skin barrier regulation. Both environmental and genetic factors result in chronic inflammation and growth of psoriatic plaques from hyper-proliferating skin cells [1].

Some medications such as lithium, beta-blockers and antimalarial treatments may trigger psoriasis, as they affect normal cell proliferation and differentiation [2]. Additionally, several environmental factors contribute to psoriasis initiation and exacerbation, including physical trauma and pharyngeal infections (streptococcal throat infections). Smoking, alcohol consumption and obesity have also been linked to the disease incidence and have been implicated in worsening of the patient’s condition, but the basis of a clear relationship between them and psoriasis does not exist [3]. Ethnicity can also affect prevalence of psoriasis, such that around 1–3% of the European population suffer from psoriasis, with prevalence varying depending on the geographical area or ethnic group studied [4]. According to the Global Psoriasis Atlas, within Great Britain, prevalence of psoriasis appears to be on the rise, from 2.3% in 1999 to 2.8% in 2013 [5], while its incidence is lower in Western Europe (1.92%) and in North Africa and the Middle East (0.57% of the total population).

Psoriasis can occur in any stage of life. However, epidemiological evidence suggests that onset occurs at two peak ages. In the UK, approximately 75% of patients have hereditary early onset psoriasis (EOP) before 16 years of age, triggered by HLA-Cw*0602 positive (Type I), which is a very aggressive type, while the remaining 25% have uninherited late onset psoriasis (LOP) after 16 years of age, which is triggered by HLA-Cw*0602 negative (type II) [6]. Psoriasis is considered a multi-factorial disease caused by hyperproliferation of keratinocytes, angiogenesis and abnormal imbalanced cells differentiation, and increased secretions of excessive pro-inflammatory mediators such as interleukins, endothelin and vascular endothelial growth factors [7]. Topical treatment of psoriasis represents the first-line treatment. However, long-term therapy induces several side effects, either local or systemic. Nanotechnology-based drug delivery systems offer a solution to overcome the limitations of conventional therapies. They have different physicochemical characters from their active constituents, such as smaller particle size or different nanoscale materials, enabling deeper penetration and localized accumulation in targeted skin layers in a controlled release manner. For example, some advanced formulations contain surfactants or permeation enhancer components so they have the ability to change the molecular structure and barrier functions of skin and make pores in tight junctions, allowing active constituents to reach deeper skin layers and improve the therapeutic outcome [8]. 

## 2. Pathogenesis of Psoriasis

Psoriasis is now known to be driven by a cluster of differentiation cells (CD 4+) and T lymphocyte helper 17 (CD4+ Th17) subset, as well as Th1 cells [9]. Th17 cells produce the cytokine interleukin 17 (IL-17) in response to the cytokine IL-23 [10]. CD8 releases several inflammatory cytokines in the skin, including IL-17, IFN-γ, IL-22 and IL-13; of these, IFN-γ is thought to promote hyperproliferation of keratinocytes in the epidermis, which results in skin thickening [11].

Meanwhile, IL-17 causes abnormal differentiation of keratinocytes and stimulates pro-inflammatory cytokines production such as IL-6 and IL-8. The increased level of vascular endothelial growth factor (VEGF) contributes to angiogenesis, dilatation, and formation of high endothelial venues, reflected as skin redness and erythema, a hallmark in psoriatic lesions. IL-1 mediates the production of IL-2 and IFN-γ by T-cells. It is also responsible for the activation of neutrophils, monocytes, eosinophils and basophils, and stimulates macrophages to synthesize tumor necrosis factor-α (TNF), IL-6 and IL-8. IL-2 triggers B cell differentiation as well as production and action of natural killer (NK) cells, monocytes and macrophages. IL-2 encourages synthesis of IFN-g, TNF, IL-6 and IL-2R, and participates in its self-production. Augmentation in blood vessels number is mediated by IL-8, which also stimulates chemotaxis and neutrophil activation. IL-15 regulates the activation, proliferation and endurance of NK cells. This interleukin also stimulates formation of new blood vessels and T cells expression of IL-17 [12]. 

**Figure 1:**
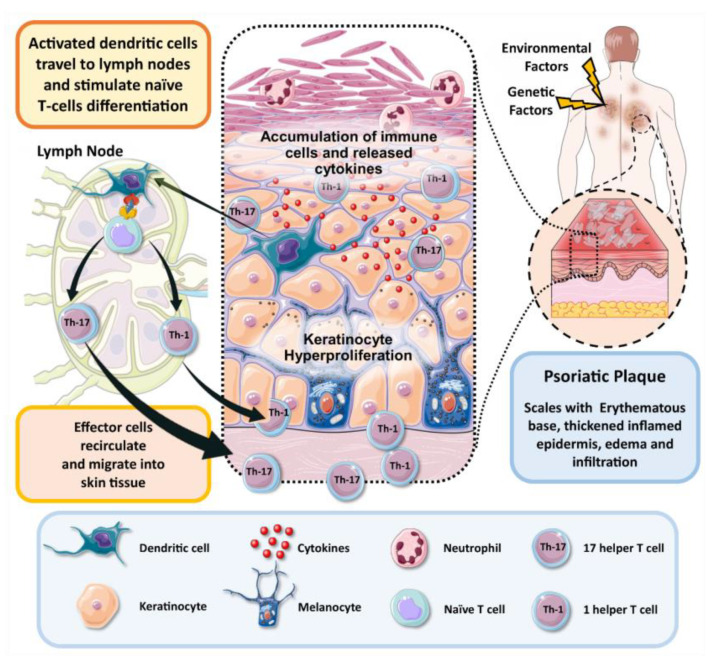
Schematic illustration of pathogenesis of psoriasis.

To sum up, T cells have a potential role in the development of psoriasis and understanding their role helps to localize the pathogenesis of disease. Th1, Th17, Treg and Th22 cells, and newly identified ‘professional’ IL-17-producing dermal CD T cells, all have significant roles in psoriasis pathogenesis. Environmental factors and pathogens activate the dendritic cells (DCs) and macrophages to release IL-23, IL-1b and other pro-inflammatory cytokines, which in turn activate the innate immunity that manifests as dermal CD T cells that produce IL17, which consecutively stimulates conventional acquired immune responses. IL-17, IL-22 and TNF-α stimulate progressively the process of keratinocytes hyperproliferation and create the inflammatory environment of disease [13]. A diagrammatic representation of the pathogenesis of psoriasis is illustrated in Figure 1.

## 3. Phenotypes of Psoriasis

One of the main subtypes of psoriasis that accounts for 90% of all cases is Psoriasis Vulgaris (PV). It is chronic plaque psoriasis (CPP) [14], clinically represented as raised itchy or painful thick red patches on the skin which are clearly defined from the non-involved skin surrounding them. These hyperproliferating patches may be covered by silver-white scales, caused by a build-up of keratinocytes, with vascular alteration that participates in enlargement of these psoriatic plaques asymmetrically. Plaques in CPP commonly appear on the outer surfaces of the knees and elbows [15].

Nail psoriasis is also one of the most prevalent types [16]. It is most commonly manifested as nail pitting, which appears as small circular areas underneath the plate, red or white in color. Other symptoms include loosening of the nail from its bed (onycholysis), discoloration due to psoriatic lesions in the nail bed (oil drop lesions), raising of the nail bed (subungual hyperkeratosis), transverse ridges (Beau’s lines), and longitudinal ridges with splitting (onychorrhexis) [17]. 

Another type is guttate psoriasis, an acute condition prevailing in the youth. Its name is derived from the Latin word “gutta” meaning “droplet”, which refers to the small round pink papules that often appear on the face, ears, and scalp. It is often thought to be triggered by a prior pharyngeal infection or tonsillitis [18].

On the other hand, psoriatic arthritis (PsA) affects approximately 6% of psoriasis patients, with a prevalence of 0.06–0.25% in the United States, 0.21% in Sweden, 0.05% in Turkey and 0.07% in Asia [19]. PsA is similar to rheumatoid arthritis, gout and reactive arthritis since it often manifests as painful inflammation in the joints and tendons, which appears shortly after the development of cutaneous psoriasis. On a cellular level, PsA is thought to be due to accumulation of particular interleukins and other inflammatory mediators in the synovial fluid [20].

## 4. Therapeutic Approaches

Pertaining to the quality of life of psoriatic patients, researchers have shown that the disease impacts their social, physical, and psychological wellbeing, with 87.8% of the patients displaying a reduced compliance with life activities in comparison to controls [21].

**Figure 2:**
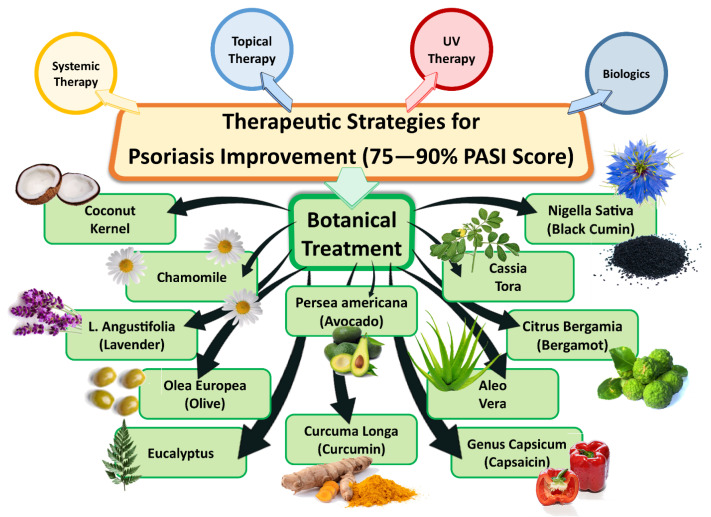
Different therapeutic approaches for alleviation of psoriasis.

Hence, the primary goal of treatment is to control the disease and its symptoms in order to enhance patients’ wellbeing, but not necessarily achieve complete healing due to relapse waves. These therapies fall into four broad categories: topical treatments, ultraviolet (UV) light therapy, traditional systemic drugs, and biologics. The efficacy of therapies is commonly measured as a percentage of patients who improved in the Psoriasis Area and Severity Index (PASI) score—a tool to measure psoriasis severity by measuring skin thickness, scales and erythema. In past studies, the primary end target was to reach an improvement in PASI score by 75%; however, this has recently been updated to a target of 90% reduction (PASI 90) [22]. Different therapeutic approaches are illustrated in Figure 2.

### 4.1. Topical Therapy

Topical therapies, which include corticosteroids, vitamin D analogues, keratolytics, coal tar, retinoids and dithranol, are considered the first line for mild/moderate cases. Currently, corticosteroids are the most commonly used line of topical therapy, because they are generally effective and well-tolerated. They act by reducing inflammation, inhibiting cell proliferation and suppression of immune cell functions, with a proven stronger curative effect than vitamin D analogues and coal tar [23]. Today, combinations of steroids and vitamin D analogues are often used; however, their prolonged use is not recommended due to the potential of adverse side effects such as skin atrophy caused by steroids and skin irritation caused by vitamin D [24].

### 4.2. UV Therapy

Phototherapy can be a narrow band of UVB or psoralen UVA (PUVA), and is often preferred in cases with large areas of widespread psoriatic plaques, resulting in longer remission period and faster healing of lesions. It works by slowing down rapid proliferation of skin cells, causing apoptosis and inhibition of immune response by the increase of regulatory T cells and depletion of pathogenic T cells and inflammatory interleukins. It participates in inhibition of activity of IL17/IL23 pathway which has a major responsibility in pathogenesis of psoriasis [25]. In the past, UVA therapy, combined with the systemic drug, psoralen, to increase sensitivity to light (PUVA), was an approach used; however, over time, the combination led to an increased risk of developing melanoma and squamous cell carcinoma of the skin. Nowadays, narrowband UVB, using only wavelengths that work for treating psoriasis, is the favored treatment regimen [26]. Monochromatic UVB therapy excimer light (308-nm) was introduced in order to increase its targeting ability and prevent unnecessary exposure of normal skin, which led to lower accumulation of dose and better safety profile. Other varieties of laser lights have also been introduced to improve the efficacy and therapeutic outcomes, including pulsed dye laser (PDL), intense pulsed light (IPL), and light emitting diode (LED) [27]. Combination of UVB therapy with bergamot oil significantly reduced the number of phototherapy sessions and UVB doses required to achieve therapeutic efficacy. This is because bergamot oil is a natural photosensitizer which contains coumarins and furocoumarins that enhance the phototherapy efficacy [28].

### 4.3. Systemic Treatments

Methotrexate, cyclosporine and acitretin are the most common systemic treatments available for psoriatic patients, with methotrexate being the most widespread drug in various cases, primarily due to its ability to target metabolism by blocking pyrimidine and purine pathways [29]. Other systemic treatments include the immune modulators, apremilast and Fumaderm. Apremilast is thought to regulate the innate immune system by blocking phosphodiesterase type 4 (PDE4), whereas the exact mechanism of Fumaderm is as-yet unclear. However, long-term suppression of the immune system endangers patients and increases risks of infection, which limits the use of systemic drugs as the first line of treatment of psoriasis. In addition, there are possible side effects for these drugs, such as the nephrotoxicity caused by cyclosporin, hepatotoxicity caused by methotrexate, making it contraindicated in any cases of existing liver illness, pregnancy and thrombocytopenia, and dyslipidemia and teratogenicity caused by acitretin [30,31].

### 4.4. Biologics

Biologics are newer, stronger drugs used in the control of psoriasis via targeting of the overactive immune system, either by inhibiting T cell functions in general or by blocking the action of pro-inflammatory mediators. They also have proved to be effective in targeting cytokines such as TNFα and IL-2/IL-23 at various stages in the psoriatic inflammatory cascade. Biologics targeting TNFα include etanercept, infliximab and adalimumab. Etanercept is a protein targeting TNF receptor by fusion, while infliximab is a monoclonal antibody that has a good primary response rate [9].

However, biological therapy can gradually lose efficacy in patients over time, for unknown reasons. A possible hypothesis is the production of neutralizing antidrug antibodies (ADAs), such as those produced in case of adalimumab, but not with etanercept [32]. Another rationale may be attributed to a decrease in serum concentration of drug in the maintenance phase of treatment, compared to the induction phase, such as what was reported in case of using infliximab [33]. 

### 4.5. Botanical Treatment

It is important to understand the potential effects of botanical agents in order to employ them for the relief of symptoms and to control disease exacerbation. In the United Kingdom, Europe and the United States, half the population of psoriatic patients need to topically apply plant-based treatments or essential oils to alleviate symptoms. Both controlled and uncontrolled clinical trials have been conducted to assess the use of topical botanical therapeutics for psoriasis. In this review, we aim to highlight essential oils/botanical agents (as shown in Figure 2) incorporated in nano-sized advanced drug delivery systems designed to target psoriasis. Examples of complementary and alternative medicinal therapies for psoriasis which are the most warranted topical plant-based therapeutics include aloe vera and capsaicin [34,35]. Topical botanical therapies have different mechanisms of action, such as inhibition of keratinocyte hyperproliferation and restoration of normal differentiation by inhibition of antimicrobial peptides (β-defensin2) and the hyper-proliferation marker keratin 17 [36]. Another mechanism is through inhibition of different signaling pathways, such as nuclear translocation of nuclear factor kappa B (NF-κB), signal transducer and activator of transcription (STAT), mitogen-activated protein kinase (MAPK), and reduction of inflammatory reactions [37]. They also have many advantages—in addition to their safety profile, they encourage better patient compliance, with fewer side effects and lower costs, making them very suitable alternative candidates to synthetic treatments [38].

#### Limitations of Botanical Treatment

However, long-term use of topical herbal remedies might be risky, due to the lack of adequately thorough clinical-based studies and safety profiles for most herbal preparations. Moreover, common adverse effects have been reported for topical plant-based medicines, namely redness, skin irritation, itching, burning and photosensitization, and sometimes, hepatotoxicity and nephrotoxicity. These are all due to the presence of additives, contaminants and mislabeled purity information, which can be a major barrier for their effective use [38]. This means that there is still a need for a standardized platform for assessment of the quality of botanical active constituents in order to fully benefit from their potential use [39].

## 5. Nano-Based Topical Drug Delivery Systems

**Figure 3:**
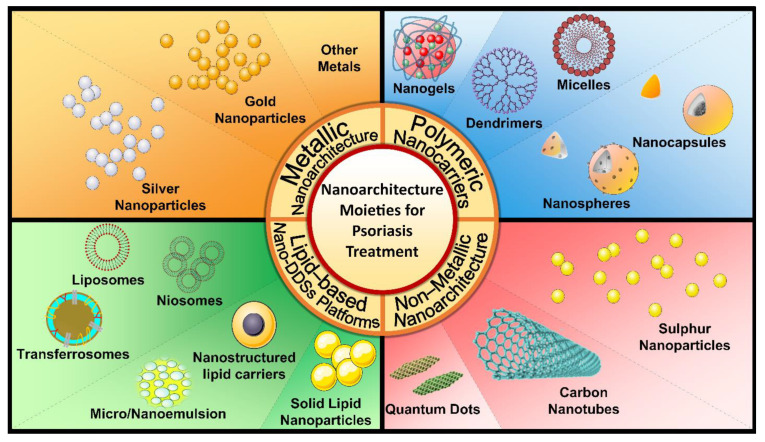
Nano-based drug delivery systems for psoriasis remediation.

Nanotechnology offers promising approaches to overcome the limitations of conventional drug delivery systems and improve the efficacy of treatment of psoriasis. Various nano-based drug delivery systems enable the delivery of the required therapeutic dose allowing drug accumulation and retention within skin layers [40]. Thus, they serve as controlled drug delivery systems, in turn reducing dosing frequency, and hence, enhancing patient compliance, as well as reducing systemic adverse effects, systemic toxicity and skin irritation. The majority of advanced delivery systems may be summarized into four main categories (Figure 3).

Lipidic systems, which comprise either a solid lipid core, mixed solid/liquid lipids, or emulsions, enable high loading of lipophilic drugs and maintain an occlusive effect which preserves close contact with the stratum cornemum (SC) upon topical application. Thereby, lipidic systems have the ability to circumvent water evaporation, increase drug permeation, and control drug release. Nanoemulsions have the advantage of being suitable vehicles for various drugs, with their ability to penetrate and interact with different skin layers, as well as a non-greasy texture. They also offer the additional advantage of providing a soothing effect, which improves inflammatory skin disease conditions such as psoriasis [41]. The most commonly utilized lipidic particulate-based carriers for the treatment of psoriasis include solid lipid nanoparticles (SLNs), nanostructured lipid carriers (NLCs), and nanoemulsions (NE). SLNs consist of a lipid core which enables deep penetration due to its small size, and accumulation in different layers of the skin, as it acts as a reservoir in the superficial layers which are the main target for psoriasis treatment [42]. NLCs are formed of a mixture of solid and liquid lipids, also at nanometer scale, which allows deep skin penetration and deposition in the targeted layers of psoriasis treatment, leading to an improvement in therapeutic efficacy and reduction of side effects [43]. NEs range from 50–200 nm, which is also an optimum size for localization in targeted skin layers and enables high entrapment efficiency with low skin irritation [8].

Lipidic vesicular systems such as liposomes, ethosomes, transferosomes, trans-ethosomes and niosomes—all of which are composed of phospholipid bilayers surrounding aqueous cores for the incorporation of hydrophilic drugs—are extensively employed in the topical treatment of psoriasis. Liposomes are vesicles ranging from 20 nm for unilamellar liposomes up to 500 nm for the multilamellar form of phospholipids, which have the ability to incorporate both lipophilic and hydrophilic drugs and reach deeper targeted skin layers due to their small size and lipidic nature allowing reduction in dose frequency and increased efficiency. Although liposomes are considered appropriate biocompatible carriers, stability problems and aggregation are major limitations to their application [44]. Niosomes are lipid vesicles composed of non-ionic surfactants and cholesterol. They bind to the skin surface, resulting in a concentration gradient which acts as the driving force for deep permeation to site-specific skin layers, hence reducing skin irritation and systematic side effects [45]. Transferosomes are deformable, elastic lipid vesicles consisting of unilamellar phospholipids and an edge activator. Transferosomes are effective in the treatment of psoriasis due to their ability to squeeze through the intercellular districts of the SC, persuaded by the transepidermal water-activity gradient resulting from occlusive application [46,47].

Polymeric drug delivery systems comprise both hydrophilic and hydrophobic copolymers in which drugs can be encapsulated or dispersed [48]. They provide high skin permeation by increasing the concentration gradient through skin layers, thus providing controlled release at targeted layers [49]. Polymeric carriers can be self-assembled as polymeric micelles with a hydrophobic core and a hydrophilic shell, which has the advantage of increasing drug entrapment and enhancing the solubility and bioavailability of drug moiety [50]. Dendrimers are highly branched monodisperse macromolecules with the drug loaded in the central core, providing targeted drug delivery systems and better permeation to deeper skin layers [51]. As for particulate polymeric systems, they mainly include nanospheres and nanocapsules. Nanospheres are polymer matrices where the drug is uniformly distributed to achieve sustained drug release by diffusion or erosion of the matrix, while nanocapsules consist of core/shell structures with the drug incorporated either inside the core or confined to the shell [52]. The main advantages of nanospheres/nanocapsules are their ability to control drug release with efficient targeting and a reduced toxicity, in addition to enhancement of stability and bioavailability of both hydrophilic and lipophilic drugs [53].

Regarding metallic nanoarchitecture, the most commonly utilized nanoparticles are gold and silver. They gained the interest of researchers in the past few years in the management of dermatological diseases mainly due to their synergistic anti-inflammatory effects, which are highly advantageous in psoriasis therapy. In addition, they accept surface functionalization and linking to different moieties, along with the ability to penetrate deeply to the skin dermis [54,55].

As for non-metallic nanosystems, carbon nanotubes, also called fullerenes, are hollow tubes less than 100 nm in size capable of deep penetration to viable skin layers, making them beneficial in the management of psoriasis [56]. By the same token, quantum dots represent a promising avenue to generate a new nanodrug delivery system with many advantages such as low toxicity, tiny size and biocompatibility, making them suitable candidates with a proved efficacy in the treatment of psoriasis [57]. Nano delivery systems are developed in order to overcome the allergic skin reactions, erythema and burning which are potential adverse effects resulting from topical application of most botanical agents, especially those relying on long-term treatments [58].

## 6. Essential Oils/Botanical Agents-Based Advanced Nano-Delivery Systems with a High Potential for the Topical Management of Psoriasis

### 6.1. Nigella sativa (Black Cumin) (NS)

*Nigella (Nigella sativa* L.) is an annual herbaceous plant belonging to the *Ranunculaceae* family [59]. It is a widely used herb that has many therapeutic indications for a variety of illnesses and conditions [60,61]. The main component of NS is black cumin oil, which consists mainly of linoleic, oleic and palmitic acids [62], in addition to thymoquinone (TQ), which has a lipophilic character and benzoquinone-like chemical structure [63].

Black cumin counteracts psoriasis mainly by inhibition of keratinocyte hyperproliferation, reduction of epidermal thickening, and abruption of abnormal differentiation. Furthermore, black cumin oil has an anti-inflammatory effect, as it inhibits pro-inflammatory mediators, hence affecting their profile and abundance. Black cumin oil also has an antioxidant effect through scavenging superoxide molecules which participate in the initiation of the inflammatory processes of psoriasis [64].

Ethosomal vesicles loaded with thymoquinone and incorporated in a hydrogel system were prepared to overcome the limitation of allergic reactions developed upon topical application. Due to their flexibility and high entrapment efficiency, they were able to permeate through the skin layers and were retained in high amounts. A reduction in psoriatic lesions and an increase in orthokeratosis also indicated enhanced antipsoriatic effects [65]. An ethanolic extract of *Nigella sativa* was assessed for its antipsoriatic activity by measuring the area of orthokeratic region in a mouse tail model, a region where cells have no nuclei and are involved in protection from UV rays and microorganisms. The mouse tail model was based on the development of granular layers from parakeratosis (orthokeratosis). The study showed that orthokeratosis was five times higher after topical application of the ethanolic extract when compared with a placebo control, which was nearly equivalent to the positive control tazarotene (0.1%) gel. The antiproliferant activity of the ethanolic extract of *Nigella sativa* was accepted and comparable to that of the positive control, asiaticoside [66].

Another clinical study was investigated for efficacy assessment of the ointment of NS oil (10% *w*/*w*) and a combination of oral capsules (300 mg) of *Nigella sativa* and 10% *Nigella sativa* ointment. Results showed excellent response and low risk of relapsing after cessation of treatment, and the proportion of patients with excellent response increased after combinatorial treatment, with lower incidence of relapse. A faster onset of action after using the new combination therapy was observed, which proved to be safe without major adverse effects, and patient compliance was also achieved as the ointment did not stain clothes and had an aromatic smell [67]. On the other hand, the poor aqueous solubility and limited skin permeation of natural bio-active thymoquinone are the main obstacles that affect its topical delivery and hinder its curative outcomes. To overcome these limiting physicochemical characters, researchers worked on the design and optimization of a nanoemulsion (NE) gel formulation for the concurrent delivery of thymoquinone, curcumin and resveratrol. The NE system consisted of oleic acid as oil phase, Tween 20 as surfactant, and PEG 200 as co-surfactant, with carbopol 940 (0.5% *w/v*) as the gelling agent. In vitro psoriatic activity was assessed using A-431 cells and results showed a high percentage of inhibition of cells proliferation. In vitro studies were supported by in vivo studies using a BALB/c mice model, which revealed that the NE showed a marked reduction in erythema, scaling and PASI score compared to the marketed formulation Betamethasone Dipropionate cream (Taro Pharmaceuticals, Inc., Hawthorne, NY, USA), as well as improved skin permeation and curative efficacy [68].

Another nanoemulsion formulation was developed, comprising NS and the antipsoriatic drug tacrolimus. A431 cell line and a BALB/c mice model were used for evaluation of in vitro and in vivo efficacy, respectively. Optimized formulation showed an increase in the local concentration of the drug in the skin with sustained release compared to the free drug in gel. Regarding in vitro efficacy, the NE in gel showed higher efficacy, expressed as a high percentage of cell growth inhibition. These results were supported by skin permeation studies which showed the highest amount of drug from the novel formulation in both SC and viable layers of skin was around five times that of the free drug in gel. In vivo studies showed that tacrolimus-loaded NE in gel caused a significant decrease in skin inflammation score and spleen average weight with a reduction in cytokine levels, indicating higher efficacy in the management of psoriasis compared to other formulations [69].

### 6.2. Bergamot

*Citrus bergamia Risso et Poiteau*, known as bergamot, is a small plant originating from the Rutaceae family, that grows naturally on the southern coast of the Calabria region of Italy, where the climate is suitable for its cultivation [70]. Bergamot essential oil (BEO) can be extracted by cold compression of the fruit, while bergamot juice (BJ) is a byproduct of the process of BEO production, obtained by squeezing the endocarp of the fruits. Many recent studies are directed to investigate the pharmacological and medical importance of bergamot oil derivatives and their use for the treatment of psoriasis. BEO has been evaluated for its potential antiproliferative effects [71], while BJ has been evaluated as an anti-inflammatory agent [72,73].

Quantification of the chemical composition of BEO has been achieved by high performance liquid chromatography–mass spectrometry (HPLC-MS), gas chromatography–mass spectrometry (GC-MS), and gas chromatography–flame ionization detection (GC-FID) techniques [74]. The main fraction (93–96%) of BEO is volatile, including borneol (about 40%), α-pinene (about 10%), and camphene (about 8%), while the residual fraction is non-volatile with phototoxicity reactions due to the presence of furanocoumarins, especially bergapten [70]. Terpenes and furanocoumarins, which include limonene, linalyl acetate (terpenes) and bergamottin (furanocoumarins), are the major components of BEO, whereas flavonoids such as naringin and neohesperidin are the main components of BJ.

The anti-inflammatory activity of BEO has been investigated using carrageenan-induced rat paw edema. It was proven to act by inhibition of IL-8 (levels of mRNA) and protein release and TNF, both of which are the main mediators of the inflammatory process of psoriasis as described previously [75]. Other studies assessed the potential anti-inflammatory activity of the polyphenolic fraction of BJ on normal human NCTC 2544 keratinocytes bare of interferon-gamma or histamine. Assays on keratinocytes proved that bergamot extracts play a role in scavenging and reduction of reactive oxygen species, which plays a vital role in the initiation and progress of skin inflammation diseases [76]. Another study investigated the therapeutic anti-inflammatory effect of BEO by topical application to a mice model to study its effect on xylene-induced ear edema. Results showed reduced mRNA expression of inflammatory mediators, i.e., IL-1β, IL-6, and TNF-α, in serum and tissues. In addition, BEO showed high permeability, which improved its therapeutic anti-inflammatory effect upon topical application; however, continuous application of BEO in high concentrations can cause skin irritation, erythema or burning, which can be overcome by using lower concentrations [77]. Other limitations of BEO application are related to phototoxicity, as BEO is rich in 5-methoxypsoralen (5-MOP) that may cause toxicity upon exposure to accidental UVA, including sunlight. It may also cause hyperpigmentation of psoriatic plaques in the neck, arms and face [78].

### 6.3. Capsaicin

Capsaicin (trans-8-methyl-N-vanillyl-6-nonenamide) is extracted from chili pepper, a plant stemming from the genus capsicum. The main active constituents of this extract are a mixture of essential oils, waxes and the colored component capsanthin, in addition to the non-volatile alkaloids, capsacinoids [79]. Capsaicin’s mechanism of action in the treatment of psoriasis depends mainly on its effect on the microvasculature of the skin. This action is likely related to its depletion of substance P from the local sensory nerve terminals and its lack of accumulation. Substance P has a main role in the pathogenesis of psoriasis by causing vasodilation and increasing blood flow to twice the normal volume [80,81]. Additionally, capsaicin was discovered to regulate the translation of hypoxia-inducible factor 1 α (HIF-1 α) through adjusting the transient receptor potential vanilloid subtype 1, thereby inhibiting hyperproliferation and maintaining the normal activities of keratinocytes and epidermal cells [82]. However, limitations of the topical administration of capsaicin arise from the induction of erythema accompanied by stinging, burning and pain sensations [9]. The use of nanotechnology for the enhancement of capsaicin effects, while reducing the associated side effects, has been recently proposed by various researchers.

Agrawal et al. aimed to overcome these limitations of topical delivery of capsaicin by encapsulation in solid lipid nanoparticles (SLNs) and nanostructured lipid carriers (NLCs), and evaluation of their potential healing effects. The optimized lipidic nanoparticles showed higher drug permeation and accumulation in the skin compared to plain capsaicin solution, with less pain sensation and itching post-topical application [83].

A mixture formulation of nanomicelles and nanoemulsions of capsaicin was developed and evaluated through imiquimod-induced psoriatic plaques. Ex-vivo skin permeation studies showed permeation of drug from the mixture and epiderimal deposition five times greater than that of the free drug [84]. Another study on an imiquimod-induced psoriasis mouse model also evaluated skin permeation, deposition and anti-inflammatory effect of the hybrid lipidic nanoparticles loaded with siRNA against tumor necrosis factor and capsaicin. Results showed significantly higher amounts of capsaicin permeated through skin layers and deposited in the SC, epidermis and dermis with a marked reduction in PASI score. Histological examination confirmed the antipsoriatic effect of the formulation and showed a normal skin condition, which was confirmed by the immunohistochemistry quantification and showed less expression of inflammatory markers, indicating the curative effect of the formulation [85].

Another study was carried out to investigate the potential effect of vesicular formulations such as liposomes, niosomes and emulsomes for achieving controlled drug release and localization of capsaicin in different viable targeted skin layers. All formulations proved high entrapment efficiency due to the lipidic nature of capsaicin, and in order to enhance its delivery, formulations were incorporated into a gel which demonstrated higher permeation amounts and flux rate compared to plain capsaicin gel. Moreover, formulations showed high skin retention in the SC and viable skin layers compared to the plain gel, with no observed skin irritation [86].

### 6.4. Cassia tora

*Cassia tora*, family *Leguminosae,* is a small annual herb grown commonly in Asia. Three flavonoids, namely luteolin1-7-O-E-glucopyranoside, quercetin-3-O-E-D-glucuronide and formononetin-7-O-E-D-glucoside, are the main constituents isolated from the ethanolic extract of the leaves. To assess the curative efficacy of these flavonoids, a UVB-induced photodermatitis animal model was designed and the histopathological analysis of the animals showed the absence of Munro’s microabscess, elongation of rete ridges, and capillary loop dilation. After oral administration of the ethanolic extract (400 mg/kg) of *C. tora*, a significant reduction in relative epidermal thickness was observed, with an inhibition in hyperproliferation of keratinocytes and a general improvement in histopathological features when compared to the positive control. These findings indicate that *C. tora* flavonoids would have an augmented activity against psoriasis [87].

Singhal and kasara investigated the effect of an o/w cream prepared with different concentrations of ethanolic extract of *C. tora* leaves up to 2 gm/kg on an ultraviolet B-induced psoriasis model in rats. The histopathological analysis showed lack of Munro’s microabscess, elongation of rete ridges, and capillary loop dilation, all of which are indicators of curative effects, in addition to a relative reduction in epidermal thickness and spleen index [88].

Moreover, in silico assessment of 15 flavonoids from *Cassia tora* was undertaken to test the feasibility of their binding to 15 anti-psoriatic targets and their binding affinity. Tumor necrosis factor-α, Bruton’s tyrosine kinase, peptidyl arginine deiminases, and spleen protein kinase were examples of the protein targets, and the docking scores showed strong binding affinities of most of the flavonoids to their psoriatic protein targets [89]. Nevertheless, the safe dose is 2000 mg/kg, and acute dermal toxicity can be reported at concentration above this level [90].

### 6.5. Aloe Vera

Aloe vera (AV) is a perennial, succulent, cactus-like plant belonging to the family *Liliaceae*. AV gel is clear and mucilaginous possessing recognized various pharmacological activities, including anti-inflammatory effects; hence, its use for various cosmetic and medicinal purposes [91,92]. AV cream has been described as an effective treatment for psoriasis without any drug-related side effects when compared to placebo. Choonhakarn carried out a prospective randomized clinical trial to compare AV gel with 0.1% triamcinolone acetonide and after eight weeks of treatment, the PASI score decreased by 7.7 in the AV group compared to 6.6 in the triamcinolone group, with no side effects. Recently, aleosin, an active constituent of AV, was found to reduce leucocytes adherence and TNF-a level, which is a major triggering factor of psoriasis pathogenesis by stimulating keratinocyte proliferation and increasing migration and adhesion of macrophages and lymphocytes [93]. In another study to assess the curative efficacy of AV on psoriasis, a group that topically applied an ointment consisting of a mixture of propolis and aloe vera was compared to the placebo group; the results showed an excellent response indicated by the disappearance of all psoriatic lesions, and normal skin appearance (no erythema, no infiltration or desquamation of skin) in 64.4% of the patients and a good response (some of the lesions disappeared) in 22% [94].

### 6.6. Avocado Oil

The main compositions of avocado oil (*Persea americana*), family *lauraceae*, are fatty acids, with oleic acid being the major one, along with carotenoids such as lutein, carotene, zeaxanthin, tocopherols, sterols and chlorophylls [95]. The anti-inflammatory action of avocado oil and its role in the treatment of psoriasis can be attributed to its catabolic action on collagen; it interferes with the metabolism of collagen by promoting its synthesis and inhibiting inflammatory infiltration [96].

A randomized prospective clinical trial using vitamin B12 cream containing avocado oil was completed to assess its efficacy in fighting plaque psoriasis over 12 weeks, in comparison to calcipotriol (vitamin D analogue) as a control drug. The anti-inflammatory effect was evaluated by a 20-MHz sonography that measured the density of corium in order to determine the degree of acanthosis, where an increase in corium density indicates less inflammatory infiltration. Results showed that calcipotriol cream reached maximum efficacy after four weeks, after which its effect began to decline, while avocado oil cream was as efficient as calcipotriol over the period of treatment, hence indicating the suitability of avocado oil cream for the long-term therapy of psoriasis [97].

### 6.7. Chamomile

Chamomila family *Asteraceae* contains various biological ingredients such as chamomile essential oil, flavonoids (apigenin, luteloin, quercetin), and terpenoids (bisabolol, chamazulene). They act by the inhibition of the production of nitric oxide, tumor necrosis factor and major inflammatory mediators synthesis such as IL1 and IL6 [98,99].

Kolahdooz et al. conducted several clinical studies to assess the efficacy of chamomile oil against plaque psoriasis. Symmetrical plaques were treated with chamomile, pumpkin oleogel and placebo, and there was an observed improvement in PASI scores, including erythema and scaling scores in chamomile and pumpkin oleogel groups compared to the placebo, with a low incidence of side effects [100]. Another study also proved that the efficacy of topical application of chamomile was equivalent to that of hydrocortisone and superior to diflucortolone, suggesting that chamomile oil could be an effective and safe treatment weapon against psoriasis and generally recognized as safe (GRAS) [101].

### 6.8. Coconut Oil

Virgin coconut oil (VCO) is a natural oil extracted from fully ripe *Arecaceae* coconut kernel [102]. VCO is composed mainly of fatty acids such as lauric, oleic, caprylic and capric acids. It has an emollient effect, which has a role in the improvement of psoriasis. In addition, it has an anti-inflammatory action, which is attributed to its hindering effect on the inflammatory mediators (cytokines) TNF-a, IFN-g, IL-6, IL-5, and IL-8, as well as an ROS scavenging property. A study conducted using a human monocyte (THP-1) model, in which cytokines production was induced by lipopolysaccharides, was used for the evaluation of VCO’s regulation of cytokines. The results, which were semi-quantitative PCR, showed a significant reduction in the levels of TNF, INF, IL6 and IL8, and a reduction of their RNA expression and protein secretion compared with dexamethasone (positive control). A human keratinocyte HaCat cells model was also used to assess the role of VCO in maintaining the barrier skin function and enabling improvement in the symptoms of psoriasis. It was observed there was an increase in the content of both involucrin and filaggrin, which are usually disturbed in psoriasis patients, in addition to skin irritation studies which proved that no adverse effects or itching were observed upon using VCO [103].

A double-blind clinical study was carried out for the assessment of VCO efficacy against dermatological lesions. VCO was compared to mineral oil; both were applied to the affected area and the results showed a significant improvement with VCO application, as 93% of patients improved, compared to 53% of patients who applied mineral oil [104].

Verallo-Rowell et al. investigated the efficacy of coconut oil in comparison with olive oil, and the inflammation score decreased by 46.8% after coconut oil application, whereas the decline stopped at 30.6% after olive oil application [105].

### 6.9. Curcumin

Tumeric is a natural polyphenolic compound obtained from the root *Curcuma longa,* a member of the ginger family, *Zingaberaceae*. Curcuminoids isolated from turmeric, which include curcumin and methoxycurcumin, are the main components, in addition to turmeric oil. In vitro studies showed that curcumin has an inhibitory effect on lipopolysaccharide and interferon-gamma-induced production of nitric oxide in macrophages [106]. It is also a strong inhibitor of the IL23/IL17 axis, which plays a significant role in the pathogenesis of psoriasis, as curcumin decreases the level of IL-17A, IL-17F, IL-22, IL-1b, IL-6 and TNF-a cytokines, as demonstrated by PCR analysis [107], in addition to preventing the proliferation and differentiation of keratinocytes [108].

An in vitro model for psoriasis was designed using imiquimod-induced differentiated HaCat cells in order to screen the activity of different curcumin concentrations (25 μM, 50 μM), and the results proved successful inhibition of proliferation of psoriasis-like cells in the model by downregulation of pro-inflammatory mediators [109].

However, poor solubility and low skin permeability are the major limitations to the use of curcumin. This is why it is essential to encapsulate curcumin into suitable carriers that can trespass the skin. A topical nanoemulsion of turmeric oil, prepared by spontaneous emulsification, was designed by Ali et al. for psoriasis treatment, and the formulation was evaluated for its physical stability, particle size and extent of irritation. Carrageenan-induced paw edema and percentage inhibition of inflammation were used to evaluate turmeric oil activity. Results showed superior activity of turmeric oil over the control (normal saline) with 70% higher inhibition in edema and no irritation post-application [108].

Curcumin was also used as an adjuvant to reduce the topical side effects of imiquimod in a nanoemulsion incorporating both curcumin and imiquimod in the oil phase. This formulation was optimized for particle size and zeta potential, and the combinatorial formulation improved symptoms of developing psoriasis like inflammation compared with the sole imiquimod gel [110].

A hydrogel of curcumin nanoemulsion was formulated and evaluated for particle size, rheological properties, skin permeation/deposition, and in vivo antipsoriatic activity. The nanoemulsion showed a level of deposition of curcumin in the skin layers seven times greater than the curcumin gel. An imiquimod BALB/c mice model was used for evaluating the antipsoriatic efficacy of the formulation, and it was shown that the nanoemulsion displayed comparable results to the positive control betamethasone, with an improvement in the symptoms of inflammation by day five of application, and complete healing after 10 days [111].

Curcumin nanoparticles were designed by Mao et al. to permeate into deeper skin layers. They were prepared using RRR-tocopheryl succinate-grafted-polylysine conjugate, which self-assembled into polymeric nanoparticles and were loaded in silk fibroin hydrogel matrix. These nanoparticles exhibited appropriate hydrodynamic diameter around 25 nm and a positive zeta potential around20, allowing strong skin-penetrating properties. Topical application of the formula on the IMQ-induced psoriasis model proved a significant decrease in the PASI score, with an attenuation in erythema, parakeratosis and hyperkeratosis. Immunohistochemical and immunofluorescent assays also established a decrease in pro-inflammatory mediators IL6 and TNF-alpha [112].

In another recent study, a nanosponge of both curcumin and caffeine was designed and optimized for the enhancement of their anti-inflammatory effects against psoriasis, and results showed a reduction in PASI score, which indicated good therapeutic efficacy of the sponge, in addition to low skin irritation [113].

### 6.10. Fish Oil

Fish oil consists of polyunsaturated fatty acids (PUFA), with alpha linoleic acid (ALA), eicosapentaeonic acid (EPA) and docosapentanenoic acid (DPA) being the most common of these PUFAs. EPA shows the inhibitory effects of lipopolysachharide-induced production of inflammatory proteins COX-2, TNF, IL-1, IL-6, IL-8 and IL-12 in various cell types, including endothelial cell monocytes, macrophages and dendritic cells. Fish oil also participates in the reduction of cytokines production, ROS, and chemotaxis of neutrophils and monocytes [114]. Metabolites of fish oil, resolvin and protectins, also cause inhibition of IL-12, superoxide production, and release of IL-23 and TNF [115].

A topical nanoemulsion of fish oil incorporated in a gel using Unitop 100 as surfactant and PEG 400 and Carbopol 971 was formulated. Results showed around 49% anti-inflammatory activity, which was enhanced to 88% by betamethasone dipropionate [116]. Topical creams containing fish oil, betamethasone dipropionate and salicylic acid showed an enhanced efficacy against psoriasis due to an increase in the of amount of betamethasone in skin layers, as well as the anti-inflammatory activity of fish oil itself. This activity of fish oil was attributed to its effect on the expression of COX-2 and the higher presence of EPA, which competes with arachidonic acid and leads to the production of less inflammatory mediators [117].

Another inflammation study using an animal model was conducted to compare the inflammatory effect of imiquimod cream and hydrogel colloidal mixture containing fish oil as antipsoriatic agent to reduce the side effects of imiquimod. The effect of fish oil was shown through measurement of transepidermal water loss and erythema of the animal model, and the scores were quite low in the imiquimod and fish oil hydrogel group compared to the imiquimod cream group [118].

### 6.11. Lavender Oil

Lavender oil (LO), an aromatic natural essential oil extracted from *Lavandula angustifolia* family *labiatae*, possesses an anti-inflammatory effect due to its two major constituents, linalool and linalyl acetate. These molecules inhibit nuclear factor kappa-light-chain-enhancer NF-kβ and MAPK activation, and reduce the level of IL-6 and TNF-α [119].

The anti-psoriatic activity of LO was measured using imiquimod-induced psoriasis in BALB/c mice, where assessment of its efficacy was based on the measurement of ear thickness, psoriasis area severity, PASI scoring and other biochemical, ELISA and histological investigations. It was shown that topical application of LO (10%) showed a significant recovery in PASI and normalization of Th17 cell-specific cytokines. It was also proven that LO reduced the levels of IL-1, IL-22, and IL-17 and improved symptoms of erythema, scaling and skin thickness. Furthermore, linalool normalized the proliferation of keratinocytes and normal parakeratosis induction [120].

### 6.12. Olive Oil

Olive oil is obtained by expression of the ripe fruit *Olea europaea*, family oleaceae. In addition to monounsaturated fat content, phenolic molecules such as oleuropein, ligstroside and oleocanthal, and their derivative phenolic alcohols such as tyrosol, are the main components of olive oil, as well as some flavonoids such as luteolin-7 glycoside and lignans [121]. All these active constituents affect inflammatory mediators involved in the immune process of psoriasis and exert ROS scavenging effects. Additionally, luteolin-7 glycoside plays an essential role in the differentiation of keratinocytes, inhibits IL22 signaling cascade, and counteracts the keratinocyte hyperproliferation effect of inflammatory mediators [122].

An herbal preparation containing a high amount (10%) of olive oil induced a curable effect of psoriasis in a case study when applied for 12 weeks, by reducing PASI more than 75% in 90 of the patients studied [123].

Another study in which olive oil was employed in the preparation of nanostructure lipid carriers (NLCs) of calcipotriol was highly promising, as olive oil possesses anti-inflammatory, keratolytic, and antioxidant properties, and increases skin hydration. Skin permeation and retention studies showed an increase in the amount of calcipotriol which resulted in a significant curative effect for the affected site of skin inflammation [124].

In another study, a nanoemulsion based on olive oil loaded with methotrexate was developed, in order to increase its therapeutic efficacy against psoriasis. The imiquimod animal model showed enhanced efficacy and reduction of psoriasis symptoms, in addition to higher drug retention in the epidermis and dermis, the target layers for the treatment of psoriasis [125].

### 6.13. Thymol

Thymol is a natural active monoterpene derived from tangerine peel in traditional Chinese medicine, family *Lamiaceae*, and commonly known as thyme and oregano. Thymol induces apoptosis of mast cells, thus reducing IgE-dependent responses, since it is a potent agonist in transient receptor potential channels, inducing calcium flux and modulating skin-mediated mast cell inflammation. Therefore, it is considered an approach for the treatment of inflammatory skin diseases such as psoriasis, as it also affects the transcription of cytokines [126].

Thymol oil was encapsulated (over 90%) in nanostructured lipid carriers (NLCs) by ultrasonication method, and then incorporated into Carbopol 940 gel, which achieved sustained release up to 18 h. Ex-vivo permeation studies showed the ability of thymol oil to affect the mobility of the SC, as monoterpene is considered a permeation enhancer, hence enabling deep penetration through thick psoriatic skin by disordering the SC integrity. Upon assessment of the formulation in a croton oil-induced inflammation model and anthralin-induced ear edema model, thymol NLCs showed higher edema inhibition than free thymol, with no signs of infiltration of neutrophils and an improvement in symptoms of erythema, scaling and skin thickness, showing comparable results to betamethasone [127].

### 6.14. Tea Tree Oil

The essential oil of *Melaleuca alternifolia*, known as tea tree oil (TTO) or melaleuca oil, is a complex mixture of terpene hydrocarbons, mainly terpenine-4-ol, terpinene, 1-8 cineole and tertiary alcohols obtained by distillation [128]. Terpenine-4-ol contributes to a decrease in the production of chemokines and cytokines (TNF, IL-1 and IL-8) due to significant reduction of CD4+ T lymphocytes production and infiltration, as proved from the biopsy of TTO-treated skin, indicating that TTO has a role in the suppression of human monocytes [129].

TTO was formulated in a nanoemulsion (5%) using polysorbate 80 as the surfactant, with isopropyl myristate and isopropyl alcohol as co-surfactants. The study showed that 14.5% of the oil permeated through the skin after 24 h of application, with no irritation to the skin [130]. Another innovative formulation of nanoemulsion used TTO as the oily phase with Tween 20 and Transcutol to incorporate clobetasol propionate. Carrageenan-induced rat paw edema was used to demonstrate the anti-inflammatory effects of the placebo, and drug-loaded nanoemulsions and results revealed inhibition of edema that was higher in the drug-loaded nanoemulsion than the placebo (84.5% versus 34%) [131]. Furthermore, both formulations showed reduction in erythema, ear thickness and edema score, and significantly reduced the protein expression of IL-12 and TNF in the dinitroflurobenzene model [132].

### 6.15. Eucalyptus Oil

Eucalyptus oil can be extracted from the leaves, fruits, buds and barks of eucalyptus tree family Myrtaceae. Its main active component is (1–8 cineole) which has been used since ancient times for its anti-inflammatory effect. The quantity of active component changes according to season and species such as *E. globuls* or *E. maidenii*. Other components of eucalyptus oil are myrcene, p-cymene and linalool [133]. Eucalyptus oil exhibits anti-inflammatory effects due to a reduction in the production of chemokines, cytokines and lipopolysaccharide mediators in the immune cells, as well as basophils, macrophages and monocytes, thus preventing activation and inhibition of mast cells degranulation. This anti-inflammatory effect was proved using a mouse IgE-mediated local allergic model, where topical application of eucalyptus oil played a significant role in reducing edema and modulating vascular permeability. Eucalyptus oil was also proven to suppress lipopolysaccharide-induced nitric oxide production in mouse macrophage cells. Consequently, from these eucalyptus oil studies, it can be deduced that it is a good candidate for the treatment of many inflammatory skin diseases through the reduction and modulation of inflammatory mediators [134]. Additionally, eucalyptus oil has remarkable antibacterial and antifungal properties, which makes it a great agent to combat such infections in the inflamed skin [135].

Examples of nano-delivery systems encapsulating natural remedies that exhibited extensive therapeutic effects in the treatment of psoriasis are summarized in Table 1.

Several clinical studies have been conducted in order to ensure the efficacy of natural remedies in the management of psoriasis, as summarized in Table 2.

## 7. Conclusions

A growing population of people and scientists are looking forward to finding the curative treatment for psoriasis, an incapacitating disease. However, controlling its manifestations and improving patients’ quality of life requires the use of different essential oils and botanical herbal agents. They act via different mechanisms in order to terminate the pathogenesis of this inflammatory disease and minimize the burdens of psoriatic plaques. By developing innovative nano-based formulations of these agents such as turmeric oil, black cumin oil and avocado oil, we can enhance their penetration through skin, improve their efficacy and accumulation in targeted skin layers, and use them as adjuvants to other established guidelines of care, especially in cases which are resistant to conventional treatment or need long-term therapy. Thus, nano-based formulations may offer a solution to overcome the limitations of topical applications of herbal botanical agents and essential oils, and challenges related to drug delivery in dermatological skin conditions. Proper choice of the type of nano-system plays an important role in their interaction with the skin. It is evident that polymeric nanoparticles, especially those having a particle size less than 100 nm and carrying a charge, are an excellent choice for the treatment of inflammatory skin disorders due to the selective accumulation in the inflamed skin [136,137,138]. On the other side, lipidic systems such as lipid nanoemulsions and nanocapsules tend to enhance the transdermal permeation of the encapsulated drugs due to their greater ability to diffuse through the different skin layers [139,140]. Therefore, polymeric nanocapsules containing natural oils would offer an excellent candidate for topical therapy. SLNs and NLCs are also known for their ability to enhance skin deposition, due to the relatively solid nature of their matrices. Such carriers would also significantly improve the stability of the vulnerable volatile oils. Many clinical trials, studies and investigations, both in vivo and in vitro, have been undertaken to assess the therapeutic efficacy and monitor adverse reactions upon topical application of these formulations. Promising results related to NE were obtained in studies of black cumin oil, tea tree oil, turmeric oil and olive oil. Other curative formulations were obtained from NLC of olive oil and thymol oil incorporated into nanogel. The decreasing of PASI and improvement of dermatological conditions were also observed through in vivo model studies of a nanosponge delivery system and ethosomal system for curcumin and thymoquinone, respectively. An albino rat model was used for the assessment of the curative efficacy of capsaicin-loaded vesicular systems, which showed promising results. However, further studies and trials are needed to thoroughly assess the potential activity and safety of these natural agents and new developments in their formulations, such as SLNs, NLCs, and nanoemulsions. Additionally, regulatory requirements and quality control guidelines are needed in order to establish a platform for standardizing herbal medicine and ensure its efficacy and safety on a large number of subjects, to achieve maximum benefit from such innovative nanotechnology delivery systems of essential oils and botanical agents against psoriasis.

## Figures and Tables

**Table 1:** Effective nano-based delivery systems encapsulating botanical agents for the treatment of psoriasis assessed via in vivo models.

Formulation	Botanical Agent (Active Constituents)	Animal Used	Results	Reference
Nanoemulsion	Tea tree oil Tepinen-4-ol	Swiss albino mice	Study showed enhancement of terpinen-4-ol permeation through skin layers with high tolerability to the formulation regarding irritation and safety	[130]
Nanoemulsion	Turmeric oil	Carrageenan induced paw edema method	Superior activity over the control group with higher stability and lack of adverse effects or irritation	[108]
Lipid-polymer hybrid nanoparticles	Capsaicin anti-TNFα siRNA	C57BL/6 mice	Improvement of psoriatic lesions and reduction of inflammatory markers compared to capsaicin solution or anti-TNFα siRNA	[85]
Mixture of nanomicelles and nanoemulsion	Capsaicin	C57BL/6 mice.	Significant reduction in PASI score and decline in spleen weight compared to free drug gel, nano-micelles, nanoemulsion	[84]
NLCs and SLNs	Capsaicin	Albino rat model	Improved drug accumulation and skin permeation with lack of irritation upon application compared to capsaicin solution	[83]
Vesicular system (liposomes, niosomes, emulsomes)	Capsaicin	Albino rat	Enhancement of skin permeation and deposition in viable skin layers for effective psoriasis treatment with minimal skin irritation compared to capsaicin plain gel	[86]
Polymeric nanoparticles incorporated in RRR-α-tocopheryl succinate-grafted-ε-polylysine conjugate (VES-g-ε-PLL) hydrogel	Curcumin	Male BALB/c mice	Enhanced permeation through skin layers and reduction of inflammatory cytokine mediators compared to curcumin	[112]
Nanoemulsion In Carbopol 840 gel	Combination of *Nigella sativa* oil and tacrolimus	BALB/c mice model	Marked decrease in skin inflammatory markers (spleen weight and cytokine level) compared to free drug in gel	[69]
NLCs incorporated in carbopol 940 gel	Thymol	Mouse model	Symptoms of psoriasis were relieved including erythema and scaling with no infiltration of neutrophils in comparison to thymol gel	[127]
Ethosomal vesicles in carbopol 934 hydrogel	*Nigella sativa* Thymoquinone	Mouse tail model	Substantial decrease in psoriatic lesions compared to *Nigella sativa* extract and plain thymoquinone suspension	[65]
Nanoemulsion	Tea tree oil (Tepinen-4-ol) and clobetasol	Carrageenan induced rat paw edema	Reduction in edema score, ear thickness and inflammatory mediators compared to placebo nanoemulsion and marketed formulation	[131]
Nanoemulsion	Curcumin	Imiquimod induced psoriasis in BALB/c mice	Significant reduction in psoriatic lesions compared to conventional imiiquimod nanogel	[110]
Nanoemulsion in carbopol 934 hydrogel	Curcumin	BALB/c mice	Improvement of symptoms and complete healing by the tenth day of topical application compared to betamethasone gel or curcumin gel	[111]
Nanosponge	Curcumin and caffeine	BALB/c mice	Improvement of PASI score and enhancement of therapeutic efficacy compared to curcumin marketed formulation	[113]
Nanoemulsion	*Nigella sativa* (NS) Thymoquinone	BALB/c mice model	Significant reduction in PASI score with reduced scaling and inflammation compared to free drug solution	[68]
NLCs enriched nanogel	Olive oil and calcipotriol	Mouse model	Curative effect and enhanced drug permeation and deposition through skin layers compared to calcipotriol enriched plain gel	[124]
Nanoemulsion	Olive oil and methotrexate	Mouse model	Better drug targeting and retention in skin layers with an enhanced efficacy against psoriasis compared to methotrexate plain gel	[125]

**Table 2:** A summary of clinical studies that assessed botanical agents/essential oils employed in the treatment of psoriasis.

Active Agent/Dosage Form	Study Type and Duration	Method	Result	Reference
*Nigella sativa* ointment and capsules	Randomized clinical trial for 12 weeks	Three groups were examined, 20 patients each: the ointment was applied to the first group, crude powder and oral capsules were given to the second group and a combination therapy of oral capsules (250 mg) and the ointment were given to the third group.	Good responses were achieved in both group 1 (65%) and group 2 (50%) with lower incidence of psoriasis relapses in more than half of the population. Results were excellent in group 3 (85%) with no observed side effects	[67]
Aloe vera cream	Prospective, randomized clinical trial for 8 weeks	80 patients were divided into two groups: the first group received aloe vera cream and the second group received triamcinolone acetonide cream. Assessment of PASI score was conducted	Significant decrease in PASI score was observed in AV cream group (7.7) compared to triamcinolone group (6.6), indicating alleviation symptoms of psoriasis	[93]
Propolis and aloe vera cream	Double-blind control study for 12 weeks	2248 patients with mild to moderate psoriasis were divided into two groups: one group received a mixture of propolis and aloe vera while the other group received placebo treatment	Symptoms of psoriasis were completely relieved/excellent response in 65% of population of propolis and aloe vera-treated group, and good response in 22% of this group, while no observed improvement in another placebo group	[94]
Avocado oil and vitamin B12 cream	Randomized, prospective clinical trial for 12 weeks	The study was conducted on 13 patients with chronic plaque psoriasis. Vitamin D3 analog (calcipotriol), avocado oil cream and vitamin B12 were applied on intraindividual right/left sides. PASI score was determined	Avocado oil cream achieved results similar to calcipotriol as PASI decreased from approximately 9 to 0.8 in both groups after 12 weeks of treatment	[97]
Chamomile oil oleogel	Double-blind, randomized clinical trial for 4 weeks	A total of 40 patients with mild-to-moderate symmetrical plaque psoriasis were treated with both placebo and chamomile oleogel. PASI score was used for clinical assessment	The mean PASI scores of the patients treated with chamomile oleogel decreased by more than 4 and were significantly lower than the placebo group	[100]
Virgin coconut oil	Randomized, double-blind, clinical trial for 8 weeks	117 patients were divided into two groups: one group received virgin coconut oil and other received mineral oil	Significant improvement was achieved in virgin coconut oil group (49%), compared to mineral oil-treated group (19%)	[104]
A mixture of black cumin, olive oil, tea tree oil, cocoa butter, vitamin A, and vitamin B12	Case series study for 12 weeks	12 patients with moderate-to-severe psoriasis were treated using the combination of the oils. PASI score was determined	Significant improvement of psoriatic symptoms in most of the cases in more than 80% of patients	[123]

## Data Availability

As no new data were created in this article, data sharing is not applicable to this article.
